# Alcohol reversibly disrupts TNF-α/TACE interactions in the cell membrane

**DOI:** 10.1186/1465-9921-6-123

**Published:** 2005-10-24

**Authors:** Kejing Song, Xue-Jun Zhao, Luis Marrero, Peter Oliver, Steve Nelson, Jay K Kolls

**Affiliations:** 1 LSUHSC Gene Therapy Program and the LSUHSC Alcohol Research Center, LSU Health Sciences Center, CSRB Rm. 601, 533 Bolivar St., New Orleans, LA 70112, USA; 2 Children's Hospital of Pittsburgh/University of Pittsburgh, Rm. 3765, 3705 Fifth Ave., Pittsburgh, PA 15213, USA

**Keywords:** Cytokines, lipopolysaccharide, inflammation

## Abstract

**Background:**

Alcohol abuse has long been known to adversely affect innate and adaptive immune responses and pre-dispose to infections. One cellular mechanism responsible for this effect is alcohol-induced suppression of TNF-α (TNF) by mononuclear phagocytes. We have previously shown that alcohol in part inhibits TNF-α processing by TNF converting enzyme (TACE) in human monocytes. We hypothesized that the chain length of the alcohol is critical for post-transcriptional suppression of TNF secretion.

**Methods:**

Due to the complex transcriptional and post-transcriptional regulation of TNF in macrophages, to specifically study TNF processing at the cell membrane we performed transient transfections of A549 cells with the TNF cDNA driven by the heterologous CMV promoter. TNF/TACE interactions at the cell surface were assessed using fluorescent resonance energy transfer (FRET) microscopy.

**Results:**

The single carbon alcohol, methanol suppressed neither TNF secretion nor FRET efficiency between TNF and TACE. However, 2, 3, and 4 carbon alcohols were potent suppressors of TNF processing and FRET efficiency. The effect of ethanol, a 2-carbon alcohol was reversible.

**Conclusion:**

These data show that inhibition of TNF-α processing by acute ethanol is a direct affect of ethanol on the cell membrane and is reversible upon cessation or metabolism.

## Background

Alcohol is a potent immunosuppressive drug and has been widely recognized for many centuries as an important risk factor for the development of infections. Among infection, pneumonia has been highly linked to alcohol abuse. In a recent case-controlled study in Spain, of 50 control subjects and 50 patients with community-acquired pneumonia, high alcohol intake was identified as a risk factor for pneumonia [1].

The cellular mechanisms by which alcohol results in immunosupression have recently been reviewed [2]. One proposed mechanism by which acute alcohol exposure suppresses innate immunity is its dose-dependent suppression of TNF-α (TNF) elaboration by mononuclear phagocytes [3-6]. The suppressive affects of alcohol on TNF elaboration occurs at several levels. Szabo and colleagues have reported decreased nuclear translocation of NF-kB and reduced steady-state levels of TNF mRNA in alcohol exposed human monocytes [3,7]. Acute alcohol exposure also results in a significant post-transcriptional and post-translational suppression of TNF production in both rodent and primate macrophages as measured by steady-state transcript levels of TNF-α mRNA [8-11]. Recently we have shown that this is in part due to the ability of acute alcohol exposure to interfere with TNF interacting with TNF-α converting enzyme (TACE), a member of the disintegrin and metalloproteinase (ADAM) family of proteins [12,13], in the cell membrane [14].

Based on these data we hypothesized that this was a direct affect of ethanol and therefore reversible when ethanol was no longer present in the cell and secondary to the ability of alcohol to intercalate in the cell membrane. If this latter condition were true, than the suppression of TNF/TACE interactions should be related to the hydrophobicity and the chain length of the alcohol. To examine these two hypotheses we used florescence resonance energy transfer (FRET) to assess TNF/TACE interactions in A549 cells as previously described [14] using clinically relevant intoxicating concentrations of ethanol, 0, 50 or 100 mM (or approximately 0, 230 or 460 mg/dl).

## Methods

### Plasmid and plasmid construction

The full-length coding sequence of human TNF-α was prepared by polymerase chain reaction (PCR) from its cDNA coded in pcDV1 (ATCC#39894) with COOH-terminal deletion using following primers: sense, 5'-AAGCTTGGTACCACCACTATGAGCACTGAAAGCATGATC-3'; antisense, 5'-TGACTAGGATCCCAGGGCAATGATTCCAAAGTAGAC-3'. The PCR product was digested with *Kpn*I and *BamH*I and inserted in-frame into p3xFLAG-CMV-14 protein expression vector (Sigma, St. Louis, MO) to form a vector: p3xFLAG-TNF-α-CMV-14 which can express a FLAG-tagged human TNF-α, driven by the CMV promoter.

### Cell Culture

A549 cells were maintained in D-MEM/F12 supplemented with 10% heat-inactivated fetal bovine serum (FBS), 2 mM L-glutamine.

### Transfection of A549 cells

Due to the complex transcriptional and post-transcriptional regulation of TNF in macrophages, to specifically study TNF processing by TACE at the cell membrane we performed transient transfections of A549 cells (which express TACE) with the TNF cDNA. A549 cells were transfected with p3xFLAG- TNF-α-CMV-14 using Lipofectamine™2000 reagent (Invitrogen) as recommended by the manufacturer. Transfected cells were incubated in the 37°C, 5% CO_2 _humidified incubator for 24 hours to get the greatest protein expression (data not shown).

Twenty-four hours post-transfection, A549 cells were split into 24-well plates, adhered for 3 hours, and randomized to treatments for 1 or 2 hours with fresh medium or medium with 50 mM or 100 mM alcohols of different one to four carbon chain lengths, methanol, ethanol, 1-propanol, and n-butanol. A subgroup of cells randomized to the 2 hour incubation time had media changed to remove the alcohol after 1 hour and cultured for an additional 1 hour washout period. Culture supernatants were then harvested for secreted TNF-α determinations by ELISA and cells were removed from culture plate by incubating with 2 mM EDTA and lysed with 1%NP-40 in phosphate-buffered saline (PBS) containing 1 mM EDTA and proteases inhibitor cocktail (Roche) to measure cell-associated TNF-α as previously described [14]. In certain experiments, RNA was extracted with Tri-zol (Invitrogen) and TNF-α mRNA elves were determined using human TNF primers and the following probe: 5'-FAM-CATCGCCGTCC-TACCAGACCAAG-Black Hole Quencher 1–3' (Biosource, Camarillo, CA). Reactions were run on an i-Cycler (Bio-RAD) and normalized per ng 18 rRNA content.

### ELISA

The levels of the cell associated form of TNF-α in cell lysates and the secreted from in culture supernatant were measured by ELISA. Before testing, both cell lysates and culture media were spun by centrifugation for 15 min at 13,000 rpm in a microfuge. ELISA was performed using kit from R&D Systems (Minneapolis, MN) and following the manufacturer's protocol. Cell-associated TNF values were normalized to total protein using a Pierce Protein assay (Pierce, Rockford, IL). Concentrations of EtOH up to 100 mM had no inhibitory effect on the TNF ELISA.

### Immunofluorescent Labeling

Transfected A549 cells were adhered to cover slips in culture. After alcohol exposure for 1 or 2 hour or after the washout period, the coverslips were washed in cold PBS to remove excess media and fixed in a 4% solution of 10% methanol-free formaldehyde (Polysciences, Inc., Warrington, PA) in PBS for 15 minutes at room temperature. After thoroughly washing the fixative off with cold PBS, the cells were blocked with 5% rabbit serum in 1% bovine serum albumin (BSA) in PBS for 15 minutes. Subsequently, the blocker was replaced with the first primary antibody, mouse anti-human TACE clone M222 (Amgen, Seattle, WA), diluted in 1% BSA at 10 ug/mL. The coverslips were thoroughly washed in three PBS changes after the 45-minute primary antibody incubation. The antibody was indirectly labeled with a rabbit anti-mouse, Cy-3 conjugated Fab fragment (Jackson Immunoresearch, West Grove, PA) at 6.5 ug/mL in 1% BSA. Next, cells were washed and blocked with 5% mouse serum for 10 minutes to saturate any open antigen binding sites within the first primary antibody. The blocker was replaced with the second, primary antibody mouse anti-human, FITC-conjugated, TNF-α clone Mab11 (BD Pharmingen, San Diego, CA) at a 10 ug/mL dilution in 1% BSA for 30 minutes. All antibody dilutions remained constant throughout experiments and were titrated to be detected at their highest saturation point, hence maximizing to an optimal donor to acceptor ratio. Finally, the coverslips were washed, mounted cell-side down in PBS on slides with spacers, and secured with nail polish.

### FRET Imaging and Data Collection

Human A549 cells mounted on coverslips were imaged using a digital fluorescence microscopy system. The system included a 12-bit, chilled charged-coupled device (CCD) Sensicam QE with a 1376 × 1040 resolution and 65% quantum efficiency at 550 nm (The Cooke Corporation, Auburn Hills, MI) on an automated Leica DMRXA microscope (Meyer Instruments, Houston, TX) using a 1.4 NA, 63 × Plan-apochromat objective. FITC and Cy3 were detected using appropriate filters (customized FITC filter cube: excitation 480/40 nm, 505 long-pass dichroic, emission 535/50; Cy3 filter cube: excitation 545/30, 565 long-pass dichroic, emission 620/60) (Chroma Technology Corporation, Brattleboro, VT). Fluorescence was excited with a 75 W Xenon arc lamp. The samples were photobleached with 100 W Mercury source. All components were controlled by Slidebook software (Intelligent Imaging Innovations, Denver, CO), which was used for capture, nearest neighbor deconvolution, and FRET analysis. Image acquisition was adjusted in milliseconds to achieve the maximum CCD camera range. As part of detecting bleed-through, typical exposure times were used to confirm no visualization of FITC-labeled samples with the Cy3 filter cube and vice versa.

All experiments were based on FRET measured by acceptor photo bleaching recovery using FITC as the donor and Cy3 as the acceptor. The Cy3 was determined to be photolabile enough to undergo the pre-measured 3-minute photo-bleaching step for a subsequent <2% initial intensity measurement. The donor had a high enough quantum yield and stability to not fade significantly. All experiments included a donor-only and acceptor-only sample to verify minimal crossover between them, hence no significant energy transfer. Data were collected for 20 different fields from a single cover slip. Each fluorescent channel was collected at six consecutive z-planes measuring 0.3 um each. The software allowed stack auto-alignment between exposures to compensate for thermally induced shifts. The FRET experiment imaging began with an initial image stack of the FITC-labeled protein (in the presence of the Cy3-labeled antibody) obtained with the FITC filter set immediately followed by an image stack captured with the Cy3 filter cube. Next, the sample was exposed to constant illumination for 3 minutes to photo bleach. An image stack of the Cy3 fluorescence after photo bleaching was then obtained followed by another FITC fluorescence image stack using the FITC filter set. The exposure times were unchanged between pre and post-photo bleaching steps. Post-FRET calculation images were deconvolved using a nearest neighbor algorithm for presentation purposes only.

### FRET data analysis

Images indicating FRET between the labeled antibodies against TNF and TACE were calculated based in the increase in donor fluorescence after acceptor photobleaching by using the following formula:

E = FITC _post photo bleach _- FITC _pre photo bleach _/ FITC _post photo bleach_

Here, E represents FRET efficiency after performing a background subtraction caused by scattered light, auto fluorescence, and dark current of the CCD camera. Fluorescence intensities were reported in arbitrary units and on a pixel-to-pixel basis. This was achieved by digitally performing the pre-photo bleaching channel subtraction from the post-photo bleaching channel (see above formula) and thresholding the difference, labeled as "mean FRET intensity". FRET efficiency data was determined by analyzing at least 50 cells per coverslip.

### Statistical methods

All data are presented as mean ± SEM. Significance was estimated using ANOVA followed by Tukey's Multiple Comparison Procedure with p < 0.05 being considered significant.

## Results

### Reversibility of Ethanol and effect of alcohol chain-length on TNF-α secretion

**Figure 1:**
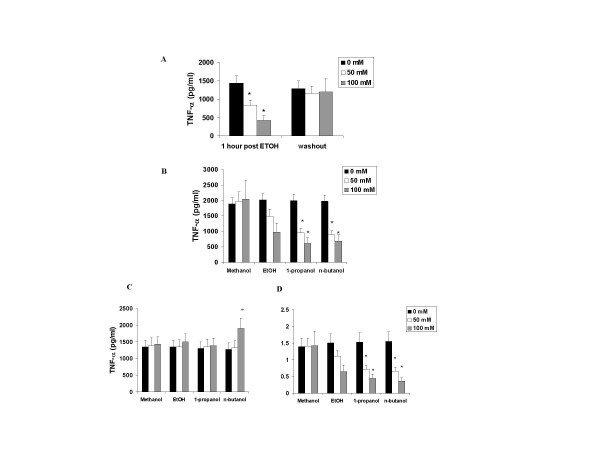
Panel A: Acute EtOH reversibly suppresses TNF-α secretion in transfected A549 cells. A549 cells transfected with the hTNF-α cDNA were re-seeded 24 hours after transfection and subjected to the addition of 0, 50 or 100 mM EtOH for 1 hour (n = 6, *. Denoted p < 0.05). For the washout experiments, a subgroup of transfected cells had media changed after one hour to fresh media without alcohol for an additional hour (n = 6, * denotes p < 0.05). Panel B: Effect of one to four carbon alcohols on TNF-α secretion (n = 4–6, * denotes p < 0.05 compared to EtOH). Panel C: Effect of one to four carbon alcohols on cell-associated TNF-α levels (n = 4–6, * denotes p < 0.05 compared to EtOH). Transfected A549 cells were treated as in Materials and Methods and TNF-α was measured in cell lysates as the cell-associated level (all data are per mg of protein). Panel D: Effect of one to four carbon alcohols on TNF processing as assessed by the ratio of shed vs. cell-associated TNF-α (n = 4–6, * denotes p < 0.05 compared to EtOH). Transfected A549 cells were treated as in Materials and Methods and TNF-α was measured in cell lysates and media and graphed as the ratio of the shed over the cell-associated level.

In pilot experiments, we determined the hourly rate of TNF secretion in transfected A549 cells and found that the rate of secretion of TNF-α was 1400–1550 pg/ml from hour 27–28 and hour 28–29 post transfection (hour 27–28 data in Figure 1A). The addition of 50 or 100 mM EtOH at 27 hours after transfection resulted in a dose-dependent secretion of TNF-α into the medium (Figure 1A). Similar suppression was also observed 2 hours after the addition of ethanol (data not shown). However, cells washed 1 hour after the addition of ethanol, and replaced with fresh media recovered their TNF secretion (Figure 1A). Mean Ct values were 22.3 + .36 per ng rRNA content for TNF message were not significantly altered by the 1 or 2 hour alcohol treatment (data not shown).

We next examined one to four carbon alcohols at concentrations of 0, 50 and 100 mM. Again we observed a dose-dependent suppression of TNF secretion by ethanol, however, methanol had no affect on TNF secretion (Figure 1B). 1-propanol and n-butanol on the other hand, were more potent than ethanol in suppressing TNF-a secretion (Figure 1B). The decrement in TNF production was not due to loss of cell viability as determined by trypan blue staining (data not shown). Moreover, this suppression was verified to occur at the level of TNF-a secretion as the alcohol with two to four carbons did not suppress cell-associated TNF concentrations (Figure 1C) and longer chain alcohols disproportionately suppressed the ratio of shed versus cell-associated TNF-a (Figure 1D), a measure of TACE efficiency.

### Effect of alcohols on TNF/TACE interactions by FRET microscopy

**Figure 2:**
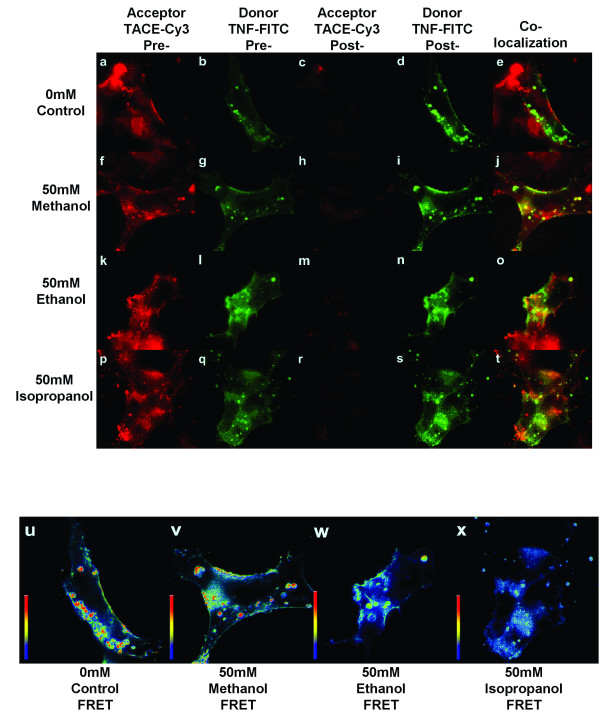
Immunofluorescent detection, co-localization, and FRET intensity in representative transfected A549 cells. Cells were stained with anti-TACE (Cy3) and anti-TNF-α (FITC) as FRET acceptor and donor, respectively (panels a-t). The staining pattern is suggestive of both cell membrane and membrane vesicle localization. In photo-bleached samples (Panels c, h, m, r) there was less than 2% initial anti-TACE (Cy3) intensity post-photobleaching. Panels u-x: FRET intensity, calculated from the difference between donor pre- and post-photobleaching intensities shown in pseudocolor.

**Figure 3:**
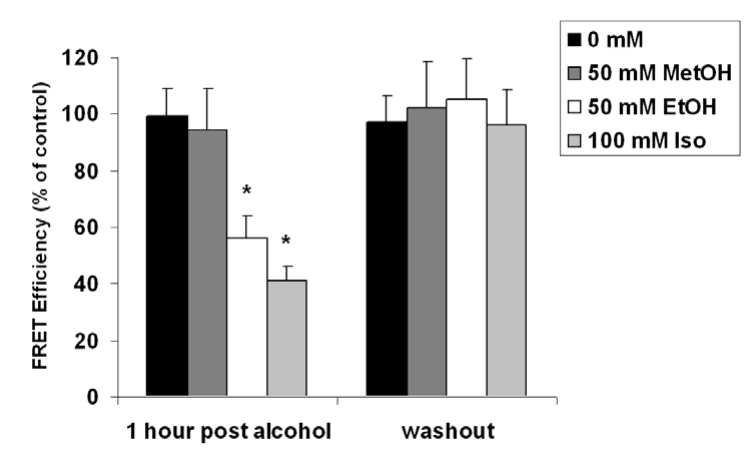
Mean FRET intensity, calculated from the difference between donor (TNF-α) pre- and post-photobleaching intensities in 20 high power fields per group (n = 4, * denotes p < 0.05 compared to 0 mM control).

To examine if the chain length of alcohol had a differential activity on TNF/TACE interactions, we performed FRET microscopy as previously described [14]. Using deconvolution microscopy, transfected A549 cells expressed TNF-α (Figure 2B) and TACE (Figure 2A). A549 cells, transfected with the human TNF cDNA, were incubated with 50 mM methanol, ethanol, or 1-propanol for 60 minutes followed by fixation in 4% methanol-free formaldehyde. Experiments were not performed with N-butanol as there was altered cell morphology (vacuolization) with this alcohol. Cells were stained for TNF and TACE and FRET efficiency was measured and calculated from 20 representative fields per group. The TACE (Cy3, acceptor) signal was photobleached (Figure 2 panels c, h, m, r) whereas the TNF (FITC) signal increased (Figure 2, panels d, I, n, s) consistent with FRET. FRET was quantified by using pixel by pixel subtraction of FITC fluorescent intensity post and pre-photobleaching. To depict FRET on the cell surface, the FRET intensity was superimposed over donor intensity as a pseudocolor image (Figure 2 panels u, v, w, and x), where blue is little or no FRET efficiency and red is peak FRET efficiency. Incubation with increasing chain lengths of alcohol resulted in an increase in cell surface sting of TNF-a (Figure 2, panels b, g, l, q) consistent with a suppression of TNF-a cleavage from these cells. This was associated with a decreased in cell surface FRET (Figure 2, panels u, v, w, and x and Figure 3). Consistent with a reversible process, FRET efficiency returned to pre-alcohol control values when the media was changed to non-alcohol containing media for an additional hour (Figure 3).

## Discussion

These studies show that acute alcohol exposure, at clinically relevant concentrations (for ethanol), block TNF secretion by posttranslational mechanism by inhibiting protein: protein interactions between TNF and TACE in the cell membrane. There have been many studies to better understand the mechanisms by which alcohol contributes to immunosuppression. Many types of infection, including bacterial pneumonia and, more recently, Hepatitis C have been shown to be adversely affected by concomitant alcohol abuse [15-18]. In addition, alcohol has been shown to be an independent risk factor for bacterial pneumonia and development of the Acute Respiratory Distress Syndrome (ARDS) [19]. In studies of pneumonia and in the setting of trauma, which is a risk factor for ARDS, blood alcohol levels can approach 50–75 mM.

One possible mechanism underlying the immunosuppressive effects of acute alcohol exposure is its effects on innate immunity. In human volunteers and experimental animal models, alcohol dose-dependently suppresses neutrophil recruitment in response to chemotactic stimuli. One mechanism operative in the lung is suppression of the pro-inflammatory cytokine, TNF, which is required for induction of CXC-chemokines critical for neutrophil recruitment [20,21], as well as expression of adhesion molecules on vascular endothelium which is required for neutrophil binding and diapedesis [20].

In addition to the known affects of ethanol on TNF-α transcription [7,22], another mechanism of ethanol-induced TNF secretion also occurs at post-transcriptional levels [11,14]. To avoid issues of transcription, and mRNA stability regulated by the TNF-α 3'-untranslated region [23,24] in these experiments, we expressed TNF under control of the heterologous CMV promoter and a poly-adenylation signal from SV40 in human bronchoalveolar cells (A549) which also express surface TACE [14].

Using protein assays and FRET microscopy, we demonstrated that acute alcohol exposure resulted in dose dependent suppression of TACE-mediated processing of TNF. Moreover the suppression of TNF secretion was recoverable within one hour of removing the ethanol. Methanol which contains a single carbon was ineffective in suppressing TNF in A549 transfectants whereas both 1-propanol (3-carbon) and n-butanol (4-carbon) were more potent in suppressing in TNF secretion. Furthermore, methanol was a weak inhibitor of TNF/TACE interactions as measured by FRET efficiency whereas both ethanol and 1-propanol potently and reversibly inhibited FRET efficiency. Taken together these data show that alcohol disrupts TNF/TACE interactions by an activity which is reversible and related to alcohol-chain length. These data support a model by which ethanol, at clinically relevant concentrations, disrupts the cell membrane in a reversible fashion, resulting in inhibition of interactions between TNF-α and TACE.

The mechanism of the reduction TNF/TACE interactions at present is unclear. The chain-length data suggest the possibility of changes in membrane fluidity [25] induced by EtOH [25] or alterations in raft compartments, or perhaps abnormal extracellular signal-regulated kinase (Erk)-mediated phosphorylation of TACE which is critical for protein kinase C-regulated TrkA cleavage [26]. Although there are no specific studies as of yet examining TNF/TACE interactions in the context of membrane rafts, members of the TNF receptor superfamily, CD40 and CD120a [27,28] as well as alpha secretases [29] have been localized to membrane rafts. TACE has been localized to non-raft compartments but depletion of cholesterol permitted the interactions of TACE with CD30 resulting in enlaced shedding of the CD30 ectodomain [30]. Both acute and chronic alcohol has been shown to increase membrane cholesterol content [31,32] and this may be one mechanism by which ethanol disrupts TNF/TACE interactions. As TACE is involved in the cleavage of multiple cytokine receptors [33,34] as well as cell adhesion molecules such as L-selectin [35,33], this may explain in part the potency alcohol as immunosuppressant of both innate and adaptive immune responses.

## Conclusion

Alcohol chain length is clearly a factor in ethanol's ability to inhibit TNF secretion as well as TNF/TACE interactions as assessed by FRET. Moreover the effect of EtOH on this process is reversible. Taken together these data strongly support a model that alcohol disrupts TNF/TACE interaction in the cell membrane.

## Competing interests

The author(s) declare that they have no competing interests.

## Authors' contributions

KS performed the transient transfections and TNF ELISA, X-J. Z. assisted with the transfections, LM did the FRET analysis, P.O., SN, and JKK assisted in experimental design and drafting the manuscript.
